# Chronic care management in primary care for patients with type 2 diabetes mellitus in Amazonas

**DOI:** 10.1017/S1463423626101005

**Published:** 2026-03-26

**Authors:** Johrdy Amilton da Costa Braga, Lucas Santos Fernandes, Maria Natália Cardoso, Elizabete Regina Araújo de Oliveira, Jhonnata Bezerra de Carvalho, Hércules Lázaro Morais Campos, Elisa Brosina de Leon

**Affiliations:** 1 Programa de Pós Graduação em Ciências do Movimento Humano da Faculdade de Educação Física e Fisioterapia, Universidade Federal do Amazonas, Manaus, Brazil; 2 Departamento de Enfermagem, Universidade Federal do Espirito Santo, Vitória, Brazil; 3 Instituto de Ciencias Exatas, Universidade Federal do Amazonas, Manaus, Brazil; 4 Instituto de Saúde e Biotecnologia, Coari, Amazonas

**Keywords:** chronic disease, health evaluation, patient care team, public health

## Abstract

**Background:**

Conducting health systems assessments helps highlight weaknesses and strengths to be explored to improve care delivery. The Assessment of Chronic Illness Care (ACIC) considers the perspective of professionals who work in the care-providing institution. Its structure comprises seven dimensions that provide specific data about the care offered. Objectives: (1) evaluate the institutional capacity for Type 2 Diabetes Mellitus (T2DM) management in the interior of the State of Amazonas from the perspective of health professionals; (2) verify the association between socio-educational, work and geographic location variables with the dimensions of the ACIC.

**Methods:**

A cross-sectional study carried out between October 2020 and December 2022 in 36 Primary Health Units (PHUs) of the seven cities of the Amazonas, totaling 230 participants. Excel 2019 and R (4.2.1) were used for data analysis. The association between independent variables and ACIC dimensions was analyzed using multiple logistic regression analysis.

**Result:**

The PHUs in the rural Amazonas have the basic capacity to care for patients with T2DM. Analysis of each dimension of the ACIC demonstrated that geographic location was the most relevant factor, showing an association with all instrument dimensions.

**Conclusions:**

Socioeducational variables showed an association with the dimensions of Decision Support, Design of the Service Delivery System, Clinical Information System, and Integration of components of the Care Model for Chronic Conditions. Work-related variables, on the other hand, were associated with the dimensions of Organization of Health Care, Community Resources, Support for Self-Management and Integration of the components of the Chronic Conditions Care Model.

## Introduction

Diabetes mellitus (DM) emerges as one of the leading public health challenges (Glovaci *et al.*, 2019). Currently, it affects approximately 536.6 million adults, and the trend is that this number will considerably increase in the next 20 years (International Diabetes Federation, 2021). Although the literature describes more than one subtype of the disease, the highest prevalence is still Type 2 Diabetes Mellitus (T2DM), accounting for 90–95% of cases (International Diabetes Federation, 2021; Sun *et al.*, 2022). In the Brazilian territory, it is estimated that T2DM has a prevalence of 9.2%, with a notable prevalence of 6.3% in the country’s northern region (Muzy *et al.*, 2021). His condition leads to various complications for diagnosed individuals, such as the onset of cardiovascular diseases, chronic kidney disease, neuropathy, and diabetic retinopathy, not to mention temporary and permanent disabilities (Zheng *et al.*, 2018). These factors cause significant economic impacts, as they increase demand in crucial sectors like healthcare and social security systems (Gillani *et al.*, 2018; Wu *et al.*, 2018; Ansari-Moghaddam *et al.*, 2020; Butt *et al.*, 2022).

This reality makes it increasingly urgent to provide adequate care to individuals with T2DM by healthcare institutions, aiming to reduce the direct and indirect damages caused by this condition. Primary Health Care (PHC) is the frontline in providing such care (BRASIL and Ministério da Saúde, 2017). PHC offers a more significant interaction between the multidisciplinary team and patients, enabling a more holistic approach (Gomes *et al.*, 2020). Conducting assessments within the scope of PHC allows for identifying existing gaps and enables planning actions to make the healthcare system more efficient (Baptista, 2017; Carvalho *et al.*, 2023).

One of the tools that can be used in this evaluation is the Assessment of Chronic Illness Care (ACIC) questionnaire (Ansari *et al.*, 2021). It considers the perspective of professionals working in the care-providing institution regarding the facility’s ability to provide effective and coordinated care for patients with chronic diseases (Antonio Filho *et al.*, 2013; Bedweyan, 2022). Its assessment allows classifying the institution’s capacity as limited, basic, reasonable, or optimal. The ACIC structure consists of seven dimensions, each providing specific information about the system. The instrument permits systematic evaluations by addressing aspects related to patient care, such as access to information, service coordination, guidance on self-care, and integration with other levels of healthcare, among others (Antonio Filho *et al.*, 2013). It can also be used as a planning tool to implement interventions to improve care (Antonio Filho *et al.*, 2013).

There are areas where geographical characteristics impose unfavourable conditions for accessing healthcare services, such as the case of the state of Amazonas, located in the northern region of Brazil (de Oliveira Lima and de Sousa, 2021). People residing in the state’s inland cities often travel long distances by small boats on waterways to receive healthcare services (Garnelo *et al.*, 2017; da Silva *et al.*, 2023). Moreover, in many locations, service quality is affected by a lack of professionals (Gama *et al.*, 2018; da Silva *et al.*, 2023). These factors can compromise providing appropriate patient support, sparking interest in understanding the current condition of healthcare services, especially the primary health units (PHUs) in these locations.

This research has two objectives: (1) evaluate the institutional capacity for Type 2 Diabetes Mellitus (T2DM) management in the interior of the State of Amazonas from the perspective of health professionals; and (2) verify the association between socio-educational, work and geographic location variables with the dimensions that make up the ACIC instrument.

## Methods

This research was motivated by the following purpose. Due to the increasing prevalence of chronic diseases driven by demographic and nutritional transitions, understanding the care process and organizing services for managing these conditions is essential. This is particularly true in regions facing significant socio-educational challenges and relying primarily on primary healthcare, such as the Amazon region. A study conducted by a research group in Manaus, the capital of Amazonas, revealed that concerning patients with hypertension, the organization of care and the institutional capacity to manage this condition were considered basic (da C. Braga *et al.*, 2022). This led us to explore the scenario in a different context – rural areas – and focus on a condition with multiple adverse health and functionality consequences.

## Study design

**Figure 1.**
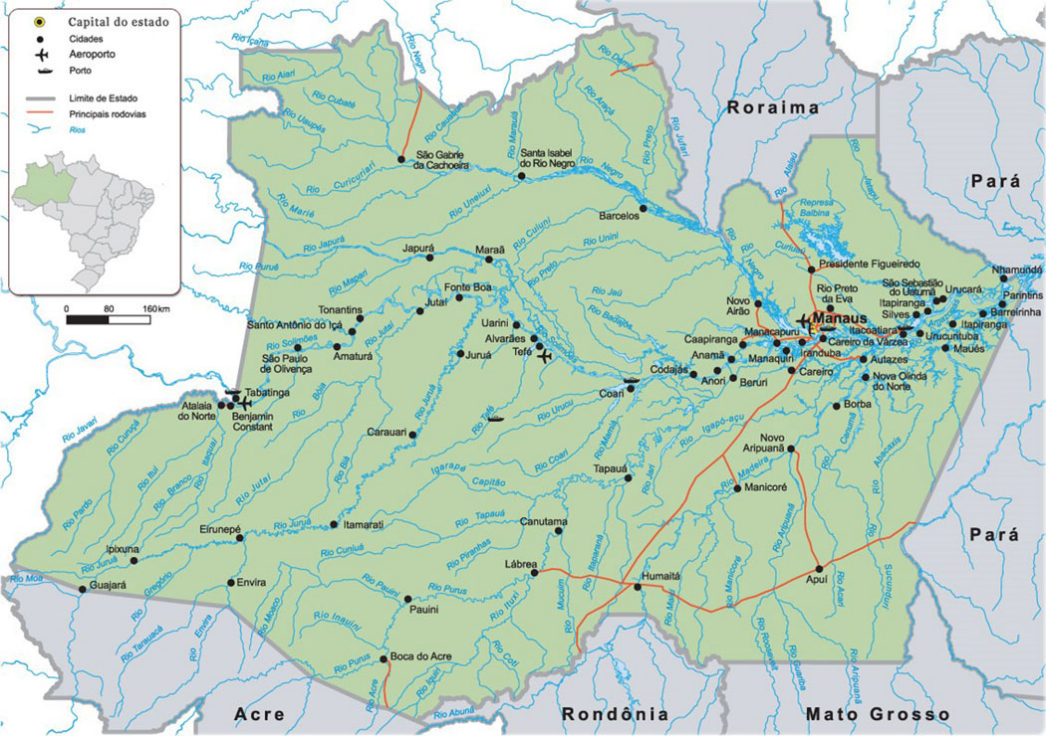
The state of Amazonas and its cities.

This investigation is characterized as a cross-sectional study with a quantitative nature. It is an integral part of a larger study titled Health in Primary Care of the Amazon Population – (SAPPA). The methodological procedures described here are based on the research protocol of this study and can be consulted by de Leon *et al.* (de Leon *et al.*, 2022). Data collection was conducted between October 2020 and December 2022 in seven cities in the interior of Amazonas: Alvarães, Coari, Iranduba, Itacoatiara, Manacapuru, Presidente Figueiredo, and Rio Preto da Eva (Figure 1).

Among the evaluated cities, Alvarães and Coari stand out for having the longest distances from the capital, 532 km and 363 km in a straight line, respectively (Distância entre as Cidades, n.d.). Access to these two cities occurs only by air and waterways, which can have an impact on development across various sectors in these areas. On the other hand, the cities of Iranduba, Itacoatiara, Manacapuru, Presidente Figueiredo, and Rio Preto da Eva are part of the metropolitan region of Manaus. This allows access by land to the capital, favouring greater development in sectors such as healthcare and technology (dos Anjos, 2018; Pimenta, 2022).

## Participants, sampling, and ethical aspects

The participants were healthcare professionals working in the PHUs of the evaluated cities. As a requirement for inclusion in the study, healthcare professionals should have a minimum of 3 months of service in the institution. Those professionals who refused to participate, those who were on leave, and those performing administrative and managerial functions were not considered. Considering the 142 PHUs distributed among the evaluated cities, setting the margin of error at 0.05 and the confidence level at 95%, a sample of 34 PHUs was obtained (de Leon *et al.*, 2022).

The study adhered to the necessary ethical procedures for its execution. Participants were requested to sign the Informed Consent Form. The Research Ethics Committee of the Federal University of Amazonas (UFAM) approved registration numbers 4,318,325 and 4,994,196 (CAAE: 25030719.4.0000.5020).

## Data collection instruments

The study data contains the ACIC instrument validated for Portuguese (Antonio Filho *et al.*, 2013), health professionals’ socio-educational and work-related information, and cities’ geographical location variables. The ACIC(Antonio Filho *et al.*, 2013) comprises 35 questions divided into seven dimensions (Table 1).

**Table 1.** Aspects inherent to the seven dimensions of the assessment of chronic illness care (ACIC)

Dimensions	Concepts
**Organization of health care**	The management of policies/programmes for chronic conditions can be more effective if the entire system (organization, institution, or unit) in which care is provided is oriented and allows more significant emphasis on care for chronic conditions.
**Community resources**	The articulation between the health system (institutions or health units) and community resources plays an important role in managing chronic conditions.
**Self-management support**	Adequate self-management support can help people with chronic conditions and their families deal with the challenges of living with and treating the chronic condition, as well as reducing health complications and symptoms.
**Decision support**	Effective management of chronic conditions ensures that health professionals have access to evidence-based information to support patient care decisions. The dimension includes guidelines and protocols based on scientific evidence, consultations with experts and health educators, and patient involvement to enable health teams to identify effective care strategies.
**Design of the service delivery system**	There is evidence that effective care management for chronic conditions involves more than simply adding interventions to a system focused on care for acute conditions. Changes in the system’s organization system’s organization and realignment of care provision are necessary.
**Clinical information system**	The dimension includes individualized information that is useful and timely for all patients with chronic conditions. It is a critical aspect of effective models of care, especially those that employ population-based approaches.
**Integration of the components of the Chronic Conditions Care Model**	Effective health systems integrate and combine all model elements, for example, associating self-care goals with records in information systems or associating local policies with activities in patients’ care plans (places for carrying out physical activities, structuring community gardens, for example).

Each ACIC dimension provides specific information about the assistance provided to healthcare service patients. Respondents assign a score ranging from 0 to 11 points for each question. This score denotes the support for chronic disease care (Antonio Filho *et al.*, 2013).

The scores for each dimension are obtained by calculating the simple arithmetic mean, considering the number of questions in each dimension. The total ACIC score is also obtained through this calculation, i.e., the simple arithmetic mean of the 35 questionnaire questions. After obtaining this total score, it is possible to classify institutional capacity for chronic conditions care. The ACIC results can be classified as follows: limited (0–2 points), basic (3–5 points), reasonable (6–8 points), and optimal (9–11 points) (Antonio Filho *et al.*, 2013).

## Data collection procedures

Initially, contact was made with the State Health Department of Amazonas (SES-AM) to obtain the initial study approval. Subsequently, each city’s Municipal Health Departments (SEMSA) were contacted to obtain the necessary study approval. After that, we agreed with the PHU coordinators to present the study and schedule the visit to the collection site. In the data agenda, researchers who had previously trained attended the PHU and, in a reserved room, participated in the research with health professionals and invited them to participate. Soon after, structured interviews began with those who agreed to participate. A data collection tool based on an open data kit (ODK) using the KoboCollect application allowed the responses to be recorded on a standard Android device (phone or tablet).

## Dependent variables

The dependent variables of the study were the dimensions of the ACIC.

## Independent variables

The independent variables were organized into the following groups: socio-educational, work-related, and geographical location.

Socio-educational variables include gender (male and female); age (18 to 29 years, 30 to 49 years, 50 years and over); marital status (single, married/in a stable relationship, divorced, widowed); educational level (primary education, secondary/technical education, higher education, other); profession (nursing, medicine, physiotherapy, dentistry, nutritionist, others); time elapsed since training (less than 1 year, 1–3 years, 4–9 years, 10–20 years, 21 years or more); postgraduate degree (none, specialization, master’s degree, doctorate, post-doctorate).

The work variables evaluated were the type of employment relationship (temporary contractor, permanent contractor), professional experience (less than 1 year, 1–3 years, 4–10 years, 10–20 years, 21 years or more), the time working at the institution (less than 1 year, 1–3 years, 4–10 years, 10–20 years, 21 years or more), and participation in actions aimed at preventing or controlling T2DM (yes and no).

The geographic location variable included the question about the municipality in which the professional lives (Alvarães, Coari, Iranduba, Itacoatiara, Manacapuru, Presidente Figueiredo, and Rio Preto da Eva).

## Statistical analysis

Data tabulation and analysis were performed using Microsoft Office Excel 2019 and R version 4.2.1, respectively. Normality was assessed using the Kolmogorov-Smirnov test. Percentages were used for categorical variables, while mean and standard deviation (SD) were used for continuous variables. The association between independent variables and ACIC dimensions was analysed using multiple logistic regression. Seven individual models were adjusted, one for each dimension. A significance level of 5% was adopted to reject null hypotheses, considering a *p*-value less than 0.05 as statistically significant. For categorical variables, the lowest level category was used as the reference. For numerical variables, the reference value was set to 0. This standard was applied across all models.

## Results

Two hundred thirty healthcare professionals from 36 PHUs participated in the study. The mean age was 36.1 ± 8.9 years. Participant characteristics are detailed in Table 2.

**Table 2.** Characterization of health professionals who work in PHC in the interior of Amazonas. Brazil. 2023. (*n* = 230)

Variables	***N***	%
**Sex**		
Male	38	16.5
Female	192	83.5
**Age range**		
18 to 29 years old	59	25.7
30 to 49 years old	154	67.0
50 years or more	17	7.4
**Marital status**		
Married/Stable union	107	47.4
Single	113	49.1
Divorced	8	3.5
Widower	2	0.9
**Education**		
Elementary school	1	0.4
High/technical education	166	72.2
University education	63	27.4
**Postgraduate studies** ^*^		
None	25	39.7
Lato Sensu Postgraduate	33	52.4
Master’s degree	2	3.2
Doctorate/Post-Doctorate	3	4.8
**Profession**		
Community health workers	124	53.9
Nursing technician	37	16.1
Nurse	27	11.7
Dentist	11	4.8
Doctor	10	4.3
Others	21	10.1
**Time elapsed since training**		
Less than 1 year	5	2.2
1 to 3 years	36	15.7
4 to 9 years	77	33.5
10 to 20 years	100	43.5
21 years or older	12	5.2
**Type of employment relationship**		
Temporary contract	215	93.4
Effective contract	15	6.5
**Professional experience**		
Less than 1 year	26	11.3
1 to 3 years	71	30.9
4 to 9 years	59	25.7
10 to 20 years	60	26.1
21 years or older	14	6.1
**Time working at the institution**		
Less than 1 year	57	24.8
1 to 3 years	85	37.0
4 to 9 years	41	17.8
10 to 20 years	42	18.3
21 years or older	5	2.2
**Participation in actions aimed at preventing or controlling T2DM**		
Yes	137	59.6
No	93	40.4
**City**		
Alvarães	24	10.4
Coari	49	21.3
Iranduba	12	5.2
Itacoatiara	38	16.5
Manacapuru	60	26.1
President Figueiredo	31	13.5
Rio Preto da Eva	16	7.0

* Analysis carried out only with professionals who have higher education.

Table 3 shows the distribution of study participants in each of the seven cities in the interior of Amazonas. The ACIC findings demonstrate that PHU’s institutional capacity in the interior of Amazonas was classified as ‘Basic’. The dimensions with the best and worst scores were ‘Design of the Service Delivery System’ and ‘Community Resources’, respectively.

**Table 3.** Distribution by city of health professionals who participated in the study (*n* = 230)

City	Profession	***N***	(%)
**Alvarães**	Community health workers	13	54.16
	Nurse	2	8.33
	Physiotherapist	2	8.33
	Doctor	2	8.33
	Nutritionist	1	4.16
	Dentist	1	4.16
	Nursing technician	3	12.5
**Coari**	Community health workers	36	73.46
	Nurse	5	10.20
	Dentist	1	2.04
	Doctor	7	14.28
**Iranduba**	Community health workers	7	58.33
	Dental assistant or oral health technician	1	8.33
	Nurse	1	8.33
	Dentist	1	8.33
	Nursing technician	2	16.66
**Itacoatiara**	Community health workers	20	52.6
	Dental assistant or oral health technician	1	2.6
	Nurse	6	15.8
	Doctor	3	7.9
	Dentist	2	5.3
	Nursing technician	6	15.8
**Manacapuru**	Community health workers	29	48.33
	Social worker	1	1.66
	Dental assistant or oral health technician	4	6.66
	Nurse	7	11.66
	Doctor	3	5
	Dentist	2	3.33
	Psychologist	2	3.33
	Nursing technician	10	16.66
	Speech therapist	1	1.66
	Social agent	1	1.66
**Presidente figueiredo**	Community health workers	14	45.16
	Social worker	1	3.22
	Dental assistant or oral health technician	4	12.90
	Nurse	3	9.67
	Doctor	1	3.22
	Dentist	3	9.67
	Nursing technician	4	12.90
	Fishing engineer	1	3.22
**Rio preto da eva**	Community health workers	4	25
	Nurse	3	18.75
	Doctor	2	12.5
	Dentist	1	6.25
	Nursing technician	5	31.25
	Pharmaceutical	1	6.25

After analysing the institutional capacity of cities in the interior of Amazonas, a multiple logistic regression test was carried out to verify the influence of some socio-educational, work, and geographic location variables on each dimension.

## Regression models

Table 5 shows the seven regression models. Only the independent variables with a significant *p*-value were presented for each model.

It is noted that geographic location was associated with all dimensions. The analysis of dimension 1 (Health Care Organization) shows that the fact that the individual has a stable union and lives in the cities of Coari, Iranduba, Itacoatiara, Manacapuru, Presidente Figueiredo and Rio Preto da Eva contributed to an increase in the score for this component. However, adequate employment and time working at the institution equal to or greater than 21 years had adverse estimated effects, impacting the score to decrease. The effects of the other associated variables on the ACIC dimensions were described in Table 5.

## Discussion

This study investigated the association between socio-educational, work-related, and geographical location variables with the dimensions comprising the ACIC instrument. When assessing the institutional capacity of the PHUs in the interior of Amazonas for providing care to patients diagnosed with T2DM, it was found that this capacity is classified as basic according to the ACIC (Table 4). This finding suggests weaknesses in the care provided to these patients, highlighting deficiencies in ensuring proactive, planned, coordinated, and person-centred care (Antonio Filho *et al.*, 2013). Seeking to provide a more detailed overview of some of the factors contributing to this situation, it was noticed that each dimension is influenced in a specific way by one or more variables.

**Table 4.** Description of the total score and the seven dimensions of the ACIC applied to health professionals in seven cities in the interior of Amazonas between 2020 and 2022 (*n* = 230)

Dimensions	Score ± SD	Classification
Organization of health care	6.8 ± 2.0 points	Reasonable
Community resources	4.6 ± 2.2 points	Basic
Self-management support	5.6 ± 2.0 points	Basic
Decision support	5.0 ± 1.8 points	Basic
Design of the service delivery system	7.2 ± 2.3 points	Reasonable
Clinical information system	5.2 ± 2.0 points	Basic
Integration of the components of the Chronic Conditions Care Model	5.3 ± 2.0 points	Basic
Total score	5.7 ± 1.6 points	Basic

## Dimension 1: Organization of healthcare

The first dimension assesses how the institution is organized and structured to care for patients with chronic diseases (Antonio Filho *et al.*, 2013). It is essential for organizing care delivery (da Costa *et al.*, 2016). This dimension was classified as ‘Reasonable’ (Table 4), and it is essential for organizing care delivery (da Costa *et al.*, 2016). The result could be attributed to an effective strategy adopted by local management and the commitment demonstrated by professionals to improve patient healthcare.

The participants’ geographical location contributed to the increase in the score in this dimension. An interesting fact about this finding is that cities in the Metropolitan Region of Manaus showed the highest estimates of positive effects. The result may be because these cities are located close to the most significant urban centre in Amazonas, meaning they have better access to resources, financing, and health infrastructure than those further away (Schor and de Santana, 2016; dos Anjos, 2018).

It was noticeable that being a permanent employee and having 20 or more years of experience in the institution negatively impacted the results. Permanent employment can lead participants to assess the institution’s structure and organization more critically, as this job security might make them more cautious. On the other hand, temporary employees lack stability and face constant uncertainties about the continuity of their employment (Dias Guimarães Junior and Barbosa da Silva, 2020; Seabra *et al.*, 2023), which might make them hesitant to point out weaknesses in their workplaces for fear of reprisals.

Twenty years or more of experience in the institution is likely related to permanent employment, which explains why these two variables show negative effect estimates for this dimension. It is noteworthy that the vast majority of professionals participating in this research have temporary contracts. Seabra *et al.* (Seabra *et al.*, 2023) reported that this type of employment relationship encourages high turnover of professionals. Additionally, longer tenure in the institution allows professionals to become familiar with its processes and practices, enabling them to recognize the strengths and weaknesses of the workplace more quickly.

## Dimension 2: Community resources

This dimension concerns the ability of the healthcare service to coordinate with community resources such as churches, schools, neighbourhood associations, and non-governmental organizations, among others (Antonio Filho *et al.*, 2013). In this investigation, this aspect proved to be the main weakness of the PHUs in the interior of Amazonas regarding the provision of care to patients with T2DM, as it had the lowest score among all dimensions. The data highlights the difficulties these PHUs face in coordinating with community resources to support and address the health needs of patients (Mendes, 2012; Veríssimo, 2020).

Analysing some of the factors contributing to this finding, it is noteworthy that the positive effect estimate generated by the participation of healthcare professionals in actions focused on T2DM prevention or control contributes to the increase in the score in this item. Participation in such actions may indicate that professionals’ engagement and search for knowledge regarding the disease can influence how they assess the need for changes in the health system, patients’ participation in care, and evaluation of results. Professionals who participate in such actions can be more prepared and updated to offer quality care to patients (Marques *et al.*, 2021; Musse, 2024).

However, almost half of the participants in this research reported not participating in such actions, which directly impacts the results found in this component. The results are related to the articulation of community resources performing better assistance (Moysés *et al.*, 2013). This fact can guide team managers and professionals to improve links with community resources (Jimenez, 2020).

Geographical location also positively influenced the score in this item. Residents of certain cities in the metropolitan area had an increase in the score. Similar to dimension 1, this could be due to the proximity to the state capital, which provides greater availability of infrastructure and access to resources.

## Dimension 3: Self-management support

This dimension is related to the support professionals provide to patients and their families to cope with the challenges posed by chronic illness and prevent complications and exacerbations (Antonio Filho *et al.*, 2013). Moysés *et al.* (2013) reported that the support offered by healthcare professionals is essential as it encourages patients to change their behaviour, empowering them to manage their care. The evaluation of this specific component demonstrated that its classification is basic. The dimension data indicates that professionals must pay more attention to patients’ needs and concerns. The findings in this component may reflect the result found in the previous item since Rodrigues *et al.* (2021) emphasized the close connection between these two dimensions. When patients receive support from the healthcare team, positive outcomes can be observed not only in the ‘Self-management Support’ dimension but also in the ‘Community Resources’ dimension (Rodrigues *et al.*, 2021).

It is important to highlight that the data collection period coincided with the COVID-19 pandemic, significantly impacting health services worldwide, including Brazil. PHC, where care for patients with T2DM is focused, has also undergone important changes. Resources and professionals were redirected to care for patients with COVID-19, which may have affected the availability of other services, such as monitoring patients with chronic diseases (Borges *et al.*, 2020; Machado *et al.*, 2023). The pandemic may have impacted the results obtained in this dimension.

The highest positive effect estimates for this dimension were observed in the geographical location variable, again focusing on cities near the capital of Amazonas. One of the reasons that can explain this finding is that this proximity to the capital facilitates professionals’ access to qualifications through workshops and in-person and online training. The proximity contributes to these individuals staying updated on matters inherent to their field, favouring, for example, a better understanding of self-management support practices (Moreira *et al.*, 2017). In more distant cities, this reality becomes more challenging due to various factors, including the region’s geographical characteristics, which hinder both access to in-person and online training (da S. Dolzane and Schweickardt, 2020; Fernandes *et al.*, 2022; Andrade *et al.*, 2023).

## Dimension 4: Decision support

This dimension defines that professionals’ access to evidence-based information is fundamental for effectively managing chronic conditions, as it provides quality assistance to patients (Antonio Filho *et al.*, 2013). It was found that this dimension also showed weaknesses. The data demonstrates the need to invest in professional qualifications in these locations. Notably, this finding aligns with what was discussed in the previous dimension regarding the difficulties in accessing qualifications faced by professionals residing in cities farther away from the capital. This fact can be observed by noting that 4 out of the five cities near Manaus showed positive effect estimates for this dimension.

As the distance from the capital increases, the positive effect estimates for this component decrease: Iranduba (38.1 km by car from the capital), Manacapuru (98.8 km by car from the capital), Presidente Figueiredo (125.5 km by car from the capital), and Itacoatiara (270 km by car from the capital). Another observation that makes this reality even more evident is that the effect estimate becomes negative for the city of Coari, contributing to the decrease in the score (Table 5). It is worth noting that this city does not belong to the metropolitan region of Manaus, and access to this location is only possible by air and water transport.

**Table 5.** Adjustment of multiple regression models with the independent variables associated with the ACIC dimensions

Dimension 1: organization of health care		
Variable	Estimated	*p*-Value
(Intercept)	5.011	<0.01
**City**		
Coari	0.823	<0.01
Iranduba	1.689	<0.01
Itacoatiara	3.427	<0.01
Manacapuru	3.080	<0.01
President Figueiredo	2.734	<0.01
Rio Preto da Eva	1.703	0.0160
**Type of employment relationship**		
Effective	−0.819	0.0468
**Marital status**	–	–
stable union	0.836	0.0259
**Time working at the institution**		
21 years or older	−2.594	<0.01

Dimension 2: community resources		
Variable	Estimated	*p*-Value
(Intercept)	2.442	<0.01
**City**		
Itacoatiara	2.402	<0.01
Manacapuru	1.962	<0.01
President Figueiredo	1.038	<0.01
Rio Preto da Eva	2.273	<0.01
**Participation in actions aimed at preventing or controlling T2DM**		
Yes	1.258	<0.01
**Time working at the institution**		
4 years to 10 years	−0.991	<0.01

Dimension 3: self-management support		
Variable	Estimated	*p*-Value
(Intercept)	3.013	<0.01
**City**	–	–
Coari	0.644	<0.01
Iranduba	3.524	<0.01
Itacoatiara	2.829	<0.01
Manacapuru	3.499	<0.01
President Figueiredo	3.477	<0.01
Rio Preto da Eva	1.709	<0.01
**Participation in actions aimed at preventing or controlling T2DM**		
Yes	0.642	<0.01

Dimension 4: decision support		
Variable	Estimated	*p*-Value
(Intercept)	4.208	<0.01
**City**		
Coari	−0.530	0.02873
Iranduba	2.598	< 0.01
Manacapuru	1.818	< 0.01
President Figueiredo	1.710	<0.01
Itacoatiara	1.583	<0.01
**Age range**		
50 years or more	−1.024	<0.01

Dimension 5: Design of the service delivery system		
Variable	Estimated	*p*-Value
(Intercept)	4.109	<0.01
**City**		
Coari	0.810	<0.01
Iranduba	3.478	<0.01
Itacoatiara	5.182	<0.01
Manacapuru	4.551	<0.01
President Figueiredo	4.288	<0.01
Rio Preto da Eva	3.778	<0.01
**Marital status**	–	–
stable union	1.188	<0.01

Dimension 6: clinical information system		
Variable	Estimated	*p*-Value
(Intercept)	3.358	<0.01
**City**		
Iranduba	1.521	<0.01
Itacoatiara	3.415	<0.01
Manacapuru	3.100	<0.01
President Figueiredo	3.044	<0.01
Rio Preto da Eva	2.559	<0.01
**Age range**		
50 years or more	−1.670	<0.01
**Time elapsed since training**		
21 years or older	1.406	<0.01

Dimension 7: Integration of the components of the chronic conditions care model		
Variable	Estimated	*p*-Value
(Intercept)	4.113	<0.01
**City**		
Itacoatiara	1.638	<0.01
Manacapuru	2.253	<0.01
President Figueiredo	0.907	<0.01
**Marital status**		
stable union	1.233	0.013
**Time elapsed since training**		
21 years or older	2.224	<0.01
**Professional experience**		
10 to 20 years	−0.865	0.026
21 years or older	−2.006	<0.01
**Time working at the institution**		
4 to 10 years	0.616	0.022
10 to 20 years	1.432	<0.01

It is also important to consider that 70% of this research sample comprised Community Health Workers and Nursing Technicians. In addition to all the difficulties already described, there is the fact that these high school professionals have even less autonomy to be absent and seek face-to-face training and qualifications in the capital when compared to higher education professionals. As for the age group of 50 years or more, it was found that this variable had an estimated negative effect. The explanation could be anchored in personal motivation. Professionals with more advanced ages are generally already preparing to complete their professional activities, leading them to no longer invest in additional qualifications (Martins and Borges, 2017). All of these particularities can influence the results of this item. Antonio Filho et al. (Antonio Filho *et al.*, 2013) emphasize that the qualification of professionals within the scope of PHC is a measure of great importance, and effective care for chronic diseases is only possible with the health team’s access to protocols and guidelines and also through interaction with specialists.

## Dimension 5: Design of the service delivery system

This element establishes that simply adding interventions to a system used to deal with acute conditions is not enough to develop effective management for the care of chronic diseases. For this to be possible, it is necessary to reorganize the health system and the provision of care (Antonio Filho *et al.*, 2013). This dimension presented the best result, standing out as the main potential. This finding aligns with the results of the first dimension analysed, which addresses the organization and structure of institutions for providing care to patients with chronic diseases. Despite the reasonable classification of these two items, the data indicate that managing care for chronic conditions is beginning to be a focus of attention in these establishments.

As happened in other components of the ACIC, the highest positive effect estimates were observed in cities in the metropolitan region of Manaus, which may also be due to greater ease of access to health resources and infrastructure. Studies indicate that the incipient use of management tools applied to the care of chronic diseases, inadequate working conditions, and the insufficient number of professionals working in institutions providing care are some of the obstacles that can make it challenging to obtain improvements in this reorganization of care (Allen *et al.*, 2011; de Paula *et al.*, 2016).

## Dimension 6: Clinical information system

It refers to the availability of clinical information that facilitates the coordination and planning of comprehensive health care for patients with chronic conditions (Moysés *et al.*, 2013). Proper implementation of this system allows the healthcare team to identify individuals with specific needs and enables the provision of care. Furthermore, it makes it possible to create an alert system for healthcare professionals and provides feedback to the healthcare team about their performance (Antonio Filho *et al.*, 2013). In this research, this dimension presented weaknesses, being classified as basic. The unavailability of electronic medical records in many locations is perhaps the main reason for this finding. With it, it is possible to integrate healthcare levels and access important information about patients, which helps in clinical decision-making, improves the quality of care, and provides safety and agility in care (Veríssimo, 2020).

According to Leal (Leal, 2014), implementing this tool often becomes challenging because it requires an adequate infrastructure from the institution. This idea may explain the results found in the regression model for this dimension, as an emphasis was observed on the geographic location variable, specifically on cities close to Manaus. As observed in the dimension related to the organization and structure of the institution, the cities in the metropolitan region of Manaus also stood out in this item, which may indicate better infrastructure and organization conditions in these locations when compared to more distant cities with more significant access difficulties. Other investigations report good results that the implementation and correct use of electronic medical records can provide, such as increased quality of care and agility in clinical diagnosis, management, and treatment of patients (Teichmann *et al.*, 2018; de A. R. Gomes *et al.*, 2019).

## Dimension 7: Integration of the components of the Chronic conditions care model

The last component defines integrating and combining all these dimensions as fundamental to an effective health system and offering adequate patient care (Moysés *et al.*, 2013). It was observed that the increased time spent assisting contributed to the decrease in the score for this dimension. Professionals with more extended experience may have more knowledge and experience to identify the specific needs of patients with T2DM compared to those with less experience. However, the increased time working at the institution led to an increase in the score for this component. This fact may be due to greater ease in accessing information, coordinating care, and working as a team, which can positively impact the quality of care for patients (Lima *et al.*, 2018).

Most of the ACIC dimensions were classified as basic, demonstrating that the PHU in the interior of Amazonas needs improvements in several aspects. Only two dimensions presented a reasonable classification. Although this finding indicates that there are potentialities, this is still not enough to ensure adequate care. It is necessary, for example, to integrate self-care goals with patients’ clinical information records or associate the articulation of community programmes with health services (Mendes, 2012; Antonio Filho *et al.*, 2013). Another measure would be to strengthen relationships between health professionals and community organizations with the participation of patients (Veríssimo, 2020). Ultimately, the integration between all components strengthens the final assessment.

## Strengths and limitations

One of the strengths of this research is that it carried out a detailed analysis of each ACIC dimension using multiple regression. This allowed us to verify the influence of different factors in the final assessment of the ability to provide care. The instrument covers various aspects inherent to the organization of health care. The inclusion of different professionals can also be highlighted in this study, as this makes it possible to obtain a broad perception of the institutional capacity of the PHU in these evaluated locations.

Among the limitations, the lack of time professionals is a barrier to participation in this research. The difficulty of applying the instrument in other municipalities due to time and financing is also a limitation. It must be considered that the associations found here do not allow causality to be determined due to the study design used.

It is important to highlight that the data collection period coincided with the COVID-19 pandemic, significantly impacting health services and people’s lives. The overload of health professionals, restrictions on access to services, and fear of contagion may have affected the participation of professionals in the research and the quality of care offered to patients with T2DM. Additionally, participants’ responses may have been influenced by the emotional context of the pandemic. Despite these limitations, these research results are relevant to identifying challenges and opportunities in organizing care for patients with T2DM in PHC.

## Conclusion

The PHUs in the interior of Amazonas presented a basic capacity to support patient management diagnosed with T2DM, according to the perspective of health professionals. When carrying out a more detailed analysis of these data, observing each of the dimensions that make up the ACIC, it was found that the geographic location variable was associated with all dimensions of the instrument. The socio-educational variables were associated with the following dimensions: Decision support, Design of the Service Delivery System, Clinical Information System, and Integration of the components of the Chronic Conditions Care Model. The variables related to work were associated with the dimensions: Organization of Health Care, Community Resources, Self-management support, and Integration of the components of the Chronic Conditions Care Model.
